# Implant-Based Versus Autologous Reconstruction After Salvage Mastectomy for Ipsilateral Breast Tumor Recurrence in the Previously Irradiated Breast: A Consecutive Institutional Series and a Proposed Autologous-First Algorithm

**DOI:** 10.3390/cancers18203409

**Published:** 2026-10-21

**Authors:** Jongmoo Park, Woosung Jang, Myeong Jae Kang, Sung Joon Kim, Byoung-Soo Ko, In Hee Lee, Joon Suk Moon, Byeongju Kang, Jeeyeon Lee, Ho Yong Park, Jeong Yeop Ryu, Kang Young Choi, Jung Dug Yang, Ho Yun Chung, Joon Seok Lee

**Affiliations:** 1 Department of Radiation Oncology, School of Medicine, Kyungpook National University, Daegu 41566, Republic of Korea; fauny11@knu.ac.kr; 2 Department of Plastic and Reconstructive Surgery, School of Medicine, Kyungpook National University, Daegu 41566, Republic of Korea; jjh2us@knuh.kr (W.J.); gabrielkld@knu.ac.kr (M.J.K.); prsryu@knu.ac.kr (J.Y.R.); kychoi@knu.ac.kr (K.Y.C.); lambyang@knu.ac.kr (J.D.Y.); hy-chung@knu.ac.kr (H.Y.C.); 3 Department of Radiation Oncology, Kyungpook National University Chilgok Hospital, Daegu 41404, Republic of Korea; ksjj216@knuh.kr (S.J.K.); bawcat@knuh.kr (B.-S.K.); 4 Department of Oncology/Hematology, Kyungpook National University Chilgok Hospital, School of Medicine, Kyungpook National University, Daegu 41566, Republic of Korea; ihleeoncology@knu.ac.kr; 5 Department of Surgery, School of Medicine, Kyungpook National University, Daegu 41566, Republic of Korea; joonsukm@knu.ac.kr (J.S.M.); libertas033@knu.ac.kr (B.K.); j.lee@knu.ac.kr (J.L.); phy123@knu.ac.kr (H.Y.P.)

**Keywords:** breast cancer, ipsilateral breast tumor recurrence, salvage mastectomy, breast reconstruction, radiotherapy, irradiated breast, breast implant, latissimus dorsi flap, treatment algorithm

## Abstract

**Background:** In postmastectomy radiotherapy, radiation reaches an already-reconstructed breast, and implant-based reconstruction remains feasible in selected patients. Far less studied is reconstruction within a previously irradiated field: after breast-conserving therapy, ipsilateral breast tumor recurrence (IBTR), and salvage mastectomy. We compared implant-based and autologous reconstruction in this setting and propose a hypothesis-generating treatment algorithm. **Methods:** From 2219 consecutive breast reconstructions (2012–2026), we cross-matched implant-removal, radiotherapy, and local-recurrence registries to identify all immediate total reconstructions of a previously irradiated breast after salvage mastectomy for IBTR (*n* = 13; 8 implant-based [all stand-alone, without vascularized flap coverage], 5 autologous [all latissimus dorsi-based]). The primary endpoint was reconstruction failure, defined in a modality-neutral manner as definitive loss or abandonment of the reconstruction; implant-based reconstructions of breasts not previously irradiated at the time of reconstruction (*n* = 689) served as a descriptive institutional reference cohort. **Results:** Seven of eight implants (87.5%) were removed at 3 to 51 months; the single retained implant has a persistent wound; none achieved complication-free success. Failure modes comprised early envelope necrosis, periprosthetic infection, and late attritional failure; chronic retained-capsule seroma followed removal without capsulectomy. All five autologous reconstructions healed without failure or unplanned reoperation (reconstruction failure, 7/8 [87.5%; 95% CI, 47.3–99.7%] vs. 0/5 [0%; 95% CI, 0–52.2%], *p* = 0.005; log-rank *p* = 0.039; median follow-up, 32.0 vs. 10.9 months; failure or major complication, 8/8 vs. 0/5, *p* = 0.0008). The implant-removal rate also exceeded that of the institutional reference cohort (87.5% vs. 11.2%; unadjusted, descriptive comparison). **Conclusions:** In this small consecutive series, stand-alone implant-based reconstruction of the previously irradiated breast was associated with a very high failure rate, whereas reconstruction importing well-vascularized autologous tissue healed in every case, a pattern biologically consistent with chronic radiation injury. These findings constitute a strong cautionary signal rather than definitive evidence. Our findings support an autologous-first approach after salvage mastectomy of the irradiated breast; stand-alone implant-based reconstruction should be offered only to carefully selected, fully counseled patients, and the proposed algorithm requires external validation.

## Simple Summary

Most women with early breast cancer are treated with breast-conserving surgery followed by radiotherapy, and the majority never require further breast surgery. When the tumor recurs in the treated breast, however, the standard treatment is removal of the entire breast (salvage mastectomy), and any reconstruction must then be performed within tissue that was irradiated years earlier. In this consecutive institutional series of immediate reconstructions after salvage mastectomy for recurrence in a previously irradiated breast, seven of eight reconstructions with breast implants were removed and none were free of major complications, whereas all five reconstructions with the patient’s own tissue healed. The implant removal rate was also considerably higher than that observed, descriptively, among implant reconstructions of breasts that had not been irradiated before reconstruction at the same institution. Although the series is small, these findings provide a strong cautionary signal; we suggest that reconstruction incorporating the patient’s own well-vascularized tissue be preferentially considered in this setting, and we propose a hypothesis-generating decision algorithm that requires external validation.

## 1. Introduction

Breast-conserving therapy, defined as breast-conserving surgery (BCS) or partial mastectomy followed by adjuvant whole-breast irradiation, is the standard of care for early breast cancer, and most patients treated in this way never require further breast surgery. A minority, however, develop ipsilateral breast tumor recurrence (IBTR) years to decades later, and the accepted oncologic salvage is total mastectomy. These patients then face reconstruction of a breast envelope that has been irradiated in its entirety, often long before.

The reconstructive literature on irradiated breasts is dominated by the postmastectomy radiotherapy (PMRT) setting, in which radiation is delivered after mastectomy and reconstruction, and in which implant-based reconstruction carries a well-documented two- to three-fold increase in complications and loss yet remains feasible in appropriately selected patients [1,2,3,4,5,6]. The salvage-after-IBTR scenario is biologically different and far less studied [7,8]: the entire skin envelope and chest wall have received therapeutic whole-breast doses, the interval from irradiation to reconstruction ranges from one year to more than two decades, and mastectomy skin flaps are raised entirely within irradiated tissue. Irradiated tissue is classically characterized as hypovascular, hypocellular, and hypoxic [9], with progressive obliterative endarteritis, fibroblast depletion, and fibrosis that permanently impair its capacity to heal [10,11,12,13,14,15]. Into such a bed, a breast implant introduces a large avascular foreign body that lowers the threshold for infection by orders of magnitude, supports biofilm formation, and depends wholly on the compromised envelope for coverage [16,17,18,19].

At our institution we observed a striking clustering of implant-based reconstruction failures in precisely this salvage population. The purpose of this study was to formally compare implant-based and autologous reconstruction of the previously irradiated breast after salvage mastectomy for IBTR, to characterize the modes and tempo of implant failure, and to propose, as a hypothesis-generating institutional approach, a reconstructive algorithm for this setting.

## 2. Materials and Methods

### 2.1. Study Design and Cohort Identification

**Figure 1:**
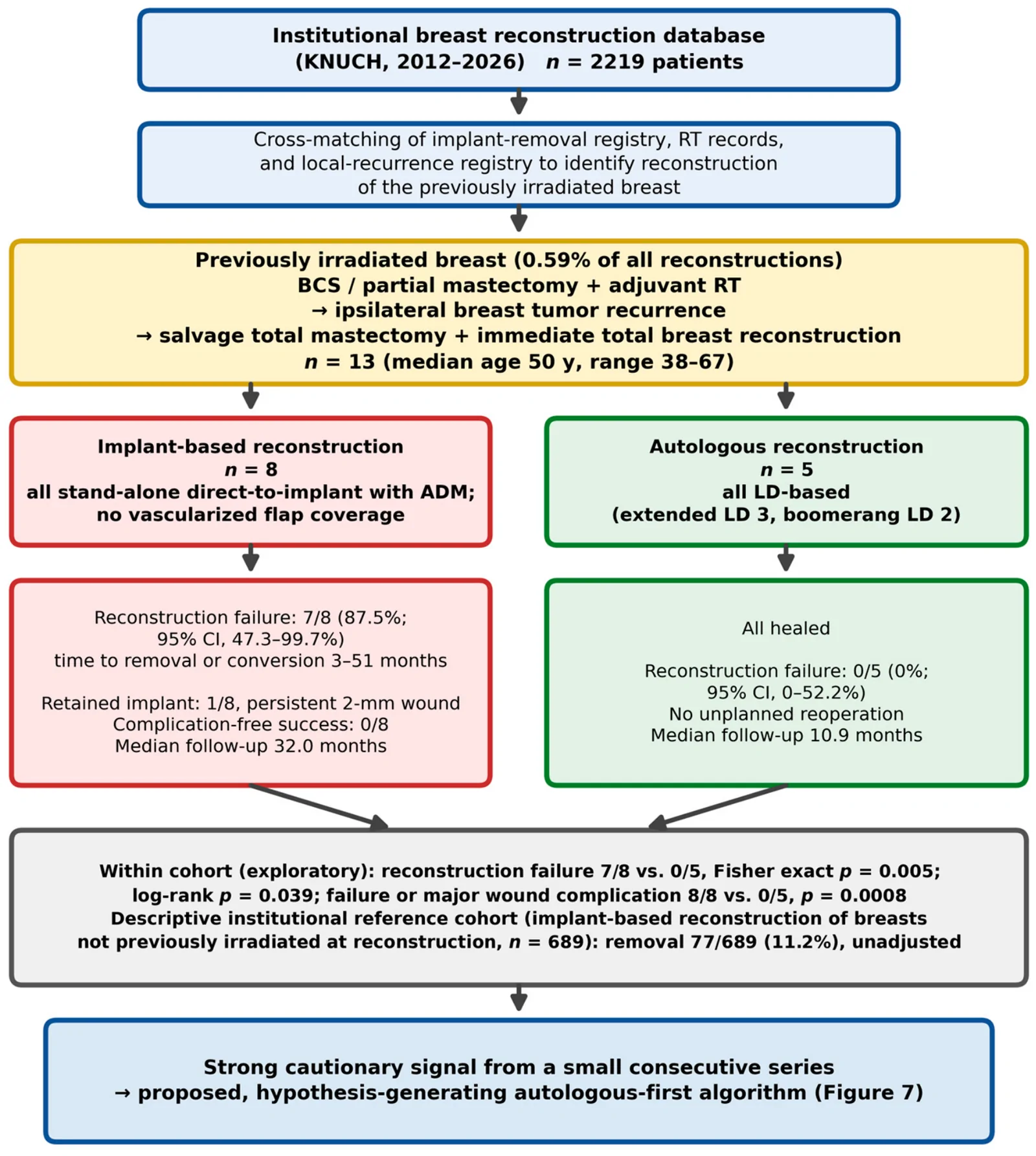
Identification of the study cohort and principal outcomes. BCS, breast-conserving surgery; RT, radiotherapy; LD, latissimus dorsi; ADM, acellular dermal matrix; CI, confidence interval; KNUCH, Kyungpook National University Chilgok Hospital.

This retrospective cohort study was approved by the institutional review board of Kyungpook National University Chilgok Hospital (approval No. KNUCH 2026-07-093), and the requirement for study-specific informed consent was waived owing to the retrospective design. In accordance with institutional practice, written consent covering the clinical use of photographs is obtained from all patients undergoing breast reconstruction. From a prospectively maintained database of 2219 consecutive patients undergoing breast reconstruction between 2012 and 2026, three institutional data sources were cross-matched. (1) A registry of all implant and expander removals (81 patients). (2) Radiotherapy treatment records (dates, institution, target volume, dose, fractionation, and boost) extracted from radiation oncology treatment archives, verified by a radiation oncologist, and supplemented by outside-hospital documentation where retrievable. (3) A hospital-wide local-recurrence registry, independent of the reconstruction database, generated by a structured keyword search of the electronic medical records (203,039 progress-note entries from 13,870 breast cancer patients) in which candidate recurrence mentions were screened with prespecified terms, filtered for negation, and adjudicated by a breast surgeon, yielding 207 patients with confirmed local recurrence; primary treatment and radiotherapy documentation for these patients were extracted from the same notes. Patients were included if they had (a) undergone BCS or partial mastectomy with adjuvant breast irradiation, (b) developed IBTR, and (c) undergone salvage total mastectomy with immediate total breast reconstruction at our institution. Thirteen consecutive patients met all criteria (Figure 1). Patients who underwent risk-reducing mastectomy after previous breast-conserving therapy without recurrence (for example, carriers of BRCA1/2 pathogenic variants) were not included because the study was designed around oncologic salvage of IBTR. All clinical variables, including the dates of reconstruction, implant removal, conversion, and reoperation, were verified against original source documents (operative records, clinical notes, and pathology reports) for every cohort patient, and the dataset was finalized before the analyses reported in this manuscript.

### 2.2. Reconstruction and Outcomes

Reconstruction was implant-based in 8 patients, all of which were direct-to-implant reconstructions with acellular dermal matrix and stand-alone prosthetic reconstructions placed beneath the irradiated mastectomy skin flaps without vascularized flap coverage at the index operation, and autologous in 5 (extended latissimus dorsi [LD] flap in 3, boomerang LD flap in 2). The primary endpoint was reconstruction failure, defined independently of modality as definitive loss or abandonment of the planned reconstruction: for prosthetic reconstruction, it was defined as definitive implant removal or unplanned conversion to flap reconstruction; for autologous reconstruction, it was defined as total flap loss, flap removal, or unplanned conversion to another reconstructive method. Partial flap necrosis was recorded as a major wound complication rather than as failure. Secondary endpoints were major wound complications (persistent wound, skin-flap necrosis, infection, chronic seroma), unplanned reoperation, and time from reconstruction to failure. Follow-up was measured from reconstruction to last follow-up (data cutoff, 28 August 2026), and time at risk for the primary endpoint from reconstruction to failure or, for reconstructions without failure, to last follow-up. Details of the reconstructive procedure (direct-to-implant versus tissue expander, implant plane, use and type of acellular dermal matrix or mesh, implant volume, mastectomy specimen weight, incision, method of mastectomy-flap perfusion assessment, and relevant intraoperative findings) and patient-level risk factors (body mass index, smoking, diabetes, systemic therapy, radiotherapy after salvage surgery, and BRCA1/2 status) for each patient were extracted from the medical records and are summarized in Supplementary Table S1 and, by reconstructive modality, in Section 3.2. Mastectomy-flap perfusion was assessed clinically (skin color and capillary refill on blanching) in all patients, and intravenous alprostadil (Eglandin; Mitsubishi Tanabe Pharma Korea, Seoul, Republic of Korea) was administered when perfusion appeared marginal.

### 2.3. Choice of Reconstructive Technique

All salvage mastectomies were performed by the institutional breast surgery team and all immediate reconstructions by the institutional plastic and reconstructive surgery team. All reconstructions were performed by a single breast reconstruction team, and each patient was managed by the same reconstructive surgeon from the index operation through all subsequent procedures and follow-up; no change in surgeon occurred in this cohort. The reconstructive method was chosen jointly by the reconstructive surgeon and the patient after counseling, without a predefined institutional protocol. Implant-based reconstruction was offered to patients whose irradiated envelope was judged clinically acceptable on preoperative examination (pliable skin without marked induration, ulceration, or chronic radiation dermatitis), whose desired breast volume was considered achievable with an implant, and who wished to avoid a donor-site scar and the longer recovery associated with flap surgery. Autologous reconstruction was selected for patients who preferred it, whose irradiated envelope appeared to be of poor quality, or who required additional skin or volume. The chronological sequence of reconstructions and modality choice is summarized in Section 3.2.

### 2.4. Institutional Reference Cohort

To provide descriptive context for the implant failure rate (rather than a matched control group), all other implant-based reconstructions performed at our institution over the same period were identified from the same database by structured query of the operative record (implant, expander, or prosthesis, including hybrid flap-plus-implant procedures; the query captured all eight study-cohort implant patients, supporting its sensitivity). After exclusion of the study cohort and of 8 patients whose breast irradiation status could not be verified, 689 implant-based reconstructions of breasts not previously irradiated at the time of reconstruction constituted the institutional reference cohort; implant removal was ascertained from the institutional removal registry, supplemented by chart review for recent removals not yet registered, and denominators and PMRT status were verified by chart review (85 removals among 705 implant reconstructions overall, 12.1%). Within the reference cohort, patients were further stratified as having received postmastectomy radiotherapy after reconstruction (PMRT, *n* = 144) or no breast irradiation (*n* = 545). The salvage cohort itself represented 13 of 2219 reconstructions (0.59%), underscoring the rarity of the scenario. Because the reference cohort differs from the salvage cohort in indication, prior treatment, tissue characteristics, reconstructive technique, and duration of follow-up, comparisons with it are presented as descriptive and unadjusted and are not intended as an estimate of the causal effect of prior irradiation. Throughout this manuscript, “not previously irradiated” refers to the irradiation status of the breast at the time of reconstruction; the PMRT subgroup received radiotherapy only after reconstruction.

### 2.5. Statistical Analysis

Given the cohort size, comparisons between reconstruction groups used the Fisher exact test with two-sided *p* values; no multivariable modeling was attempted. Given the sample size, all *p* values are regarded as exploratory. Baseline characteristics of the two reconstruction groups are presented descriptively, without formal hypothesis testing. Continuous variables are reported as median (range). Proportions are reported with exact (Clopper–Pearson) 95% confidence intervals (CIs), and differences in proportions with Newcombe hybrid score 95% CIs; because no failure occurred in the autologous group, ratio measures were not estimated for the comparison between modalities. Freedom from reconstruction failure was estimated with the Kaplan–Meier method, with reconstructions without failure censored at last follow-up and 95% CIs for the cumulative failure probability calculated on the log–log scale with Greenwood’s variance, and was compared between modalities with the log-rank test. Because most implant failures occurred within the first postoperative year and the most recent reconstructions had a shorter follow-up, a sensitivity analysis was restricted to patients with at least 12 months of follow-up. Statistical analyses were performed with IBM SPSS Statistics, version 27.0 (IBM Corp., Armonk, NY, USA); confidence intervals were calculated with the statsmodels library in Python (version 3.12; Python Software Foundation, Wilmington, DE, USA).

## 3. Results

### 3.1. Local-Recurrence Landscape at the Institution

Physician adjudication of the text-mined registry confirmed 207 patients with documented local recurrence (median age at diagnosis, 51 years; range, 27–89). This registry was derived from the hospital-wide breast cancer population (13,870 patients) rather than from the reconstruction database; the great majority of these 207 patients never underwent reconstruction, and the two data sources overlap only where a recurrence proceeded to salvage reconstruction. The recurrence was local only in 177 patients (85.5%), local with regional disease in 18 (8.7%), and local with distant disease in 12 (5.8%). Note-based abstraction identified a breast-conserving primary operation in at least 88 patients and a mastectomy-type primary in at least 91, and documented prior breast irradiation in at least 107 (51.7%; explicitly absent in 10, undocumented in the remainder). Therefore, at a minimum, 50 patients experienced recurrence in a previously irradiated, conserved breast, which is the population addressed by this study. Of these, seven (14.0%) underwent immediate total breast reconstruction at salvage mastectomy at our institution and are included in the present cohort; the remaining six cohort patients had received their primary treatment, including irradiation, at outside hospitals before referral.

### 3.2. Cohort Characteristics

The 13 patients had a median age of 50 years (range, 38–67) at salvage reconstruction. Primary treatment was BCS or partial mastectomy with adjuvant whole-breast irradiation. Radiotherapy records were documented in all thirteen patients; the course preceding salvage surgery was delivered between 1999 and 2024: regimens ranged from conventional fractionation (50–50.4 Gy in 25–28 fractions) to hypofractionated schedules (42.5–42.6 Gy in 16 fractions and 40 Gy in 15 fractions with simultaneous integrated boost), with a tumor-bed boost of 8–16 Gy in twelve patients and no boost in one, for a total dose of 48–66 Gy (median: 60 Gy). Salvage mastectomy (nipple- or skin-sparing in the majority) with immediate reconstruction was performed between 2022 and 2026. Patient-level details are given in Table 1. Baseline characteristics by reconstructive modality are compared descriptively in Table 2. The groups were broadly similar in age, body mass index (median: 22.0 vs. 24.7 kg/m^2^), and the use of a tumor-bed boost; the autologous group had received a somewhat higher median total dose (60.0 vs. 53.6 Gy), whereas hypofractionated whole-breast regimens (4/8 vs. 1/5) and nipple-sparing mastectomy (7/8 vs. 1/5) were more common in the implant group. The two modalities were interspersed over the study period rather than clustered: implant-based reconstruction was performed in 2022 (*n* = 1), 2023 (*n* = 3), 2024 (*n* = 1), 2025 (*n* = 2), and 2026 (*n* = 1), and autologous reconstruction was performed in 2023 (*n* = 2), 2025 (*n* = 1), and 2026 (*n* = 2) (Table 1). All eight implant reconstructions were direct-to-implant with acellular dermal matrix, and two followed bilateral salvage mastectomy. Seven of the eight implants were placed prepectorally and one subpectorally. Smoking and diabetes were each present in one patient, both in the implant group. One patient in each group had a documented pathogenic variant (BRCA1 in patient 11, implant group; BRCA2 in patient 7, autologous group). The interval from radiotherapy to salvage reconstruction was a median of 5.5 years (range, 1–24) in the implant group and 7 years (range, 4–19) in the autologous group (Table 1 and Table 2). Median follow-up after reconstruction was 32.0 months (range, 6.4–52.1) in the implant group and 10.9 months (range, 6.8–37.1) in the autologous group; the three reconstructions performed in 2026 (patients 1 and 8, autologous; patient 10, implant) had less than 8 months of follow-up (Table 1).

### 3.3. Implant-Based Reconstruction

**Figure 2:**
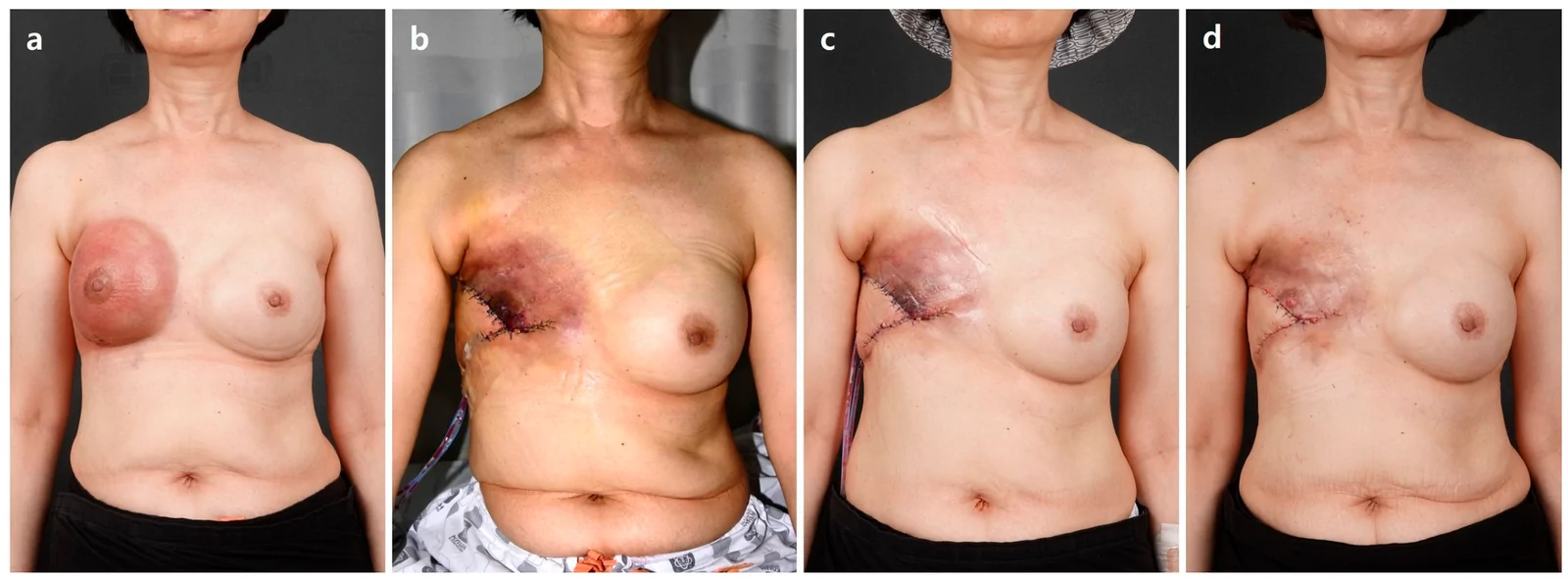
Clinical course of a late implant failure with persistent envelope inflammation. (**a**) Preoperative appearance at postoperative month 51: persistent erythema, pain, and tightness of the reconstructed breast despite prolonged multidisciplinary management, including allergy consultation. The patient declined re-reconstruction. (**b**) Immediately after implant explantation and capsulectomy. (**c**) Six days and (**d**) twelve days after explantation: progressive resolution of inflammation and settling of the envelope.

**Figure 3:**
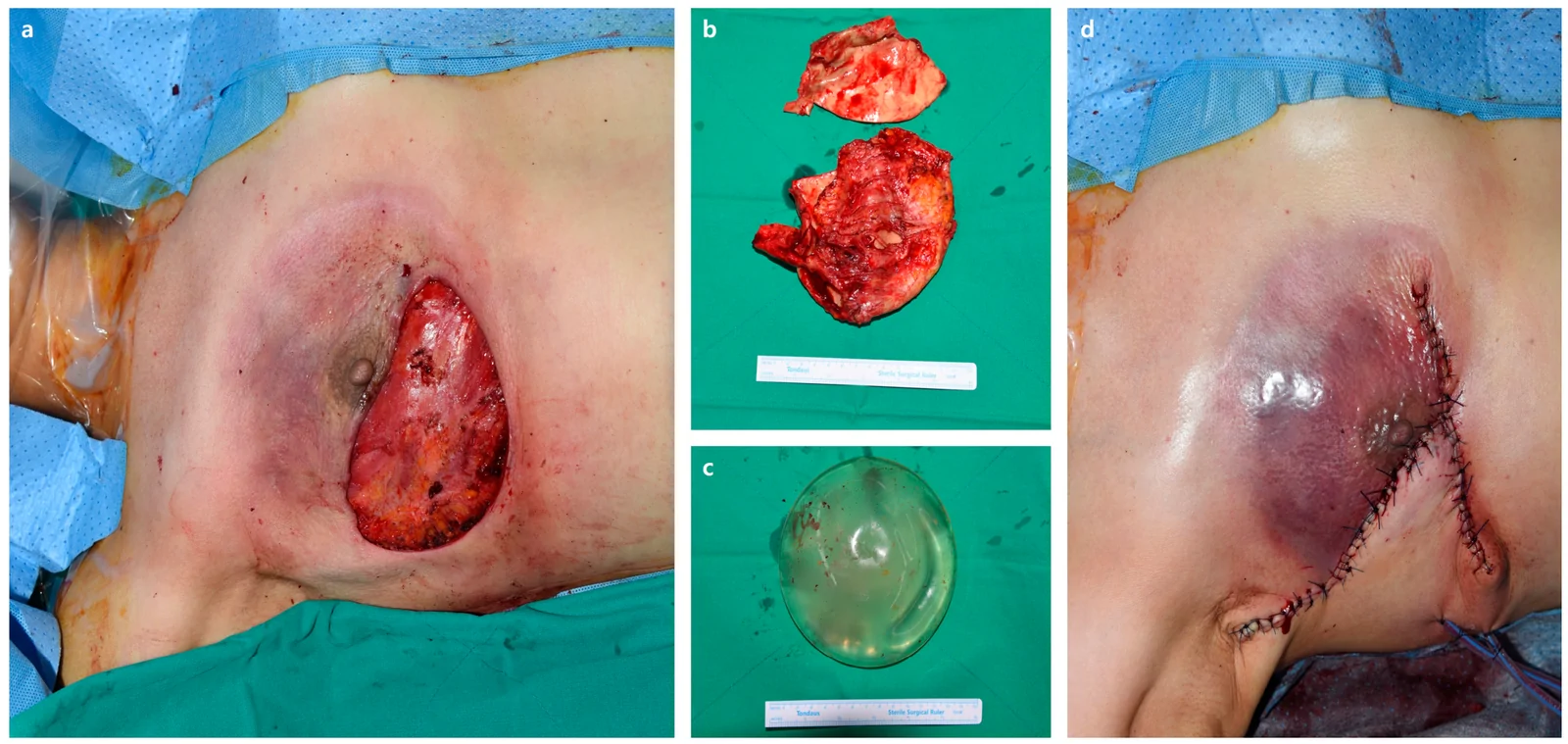
Explantation and capsulectomy in the same patient at postoperative month 51. (**a**) Intraoperative view through the lateral inframammary access used for the original endoscopic mastectomy, exposing the most erythematous zone of the envelope. (**b**) The excised capsule. (**c**) The explanted implant. (**d**) Closure incorporating a local rotation flap.

**Figure 4:**
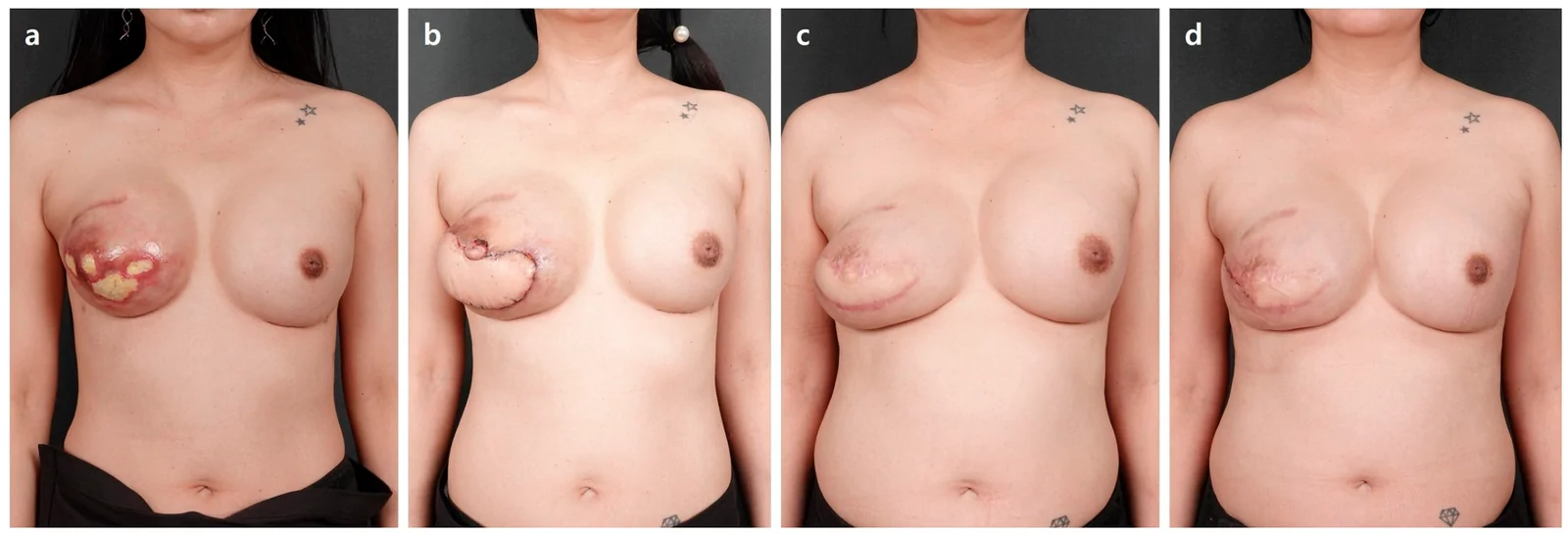
Salvage conversion after implant failure with extensive necrosis. (**a**) Postoperative month 3: extensive skin-flap necrosis over the failed implant-based reconstruction. (**b**) Postoperative month 9, after conversion to latissimus dorsi flap transfer with implant. (**c**) Postoperative month 12: healed reconstruction before flap debulking. (**d**) Postoperative month 12, after the debulking procedure.

**Figure 5:**
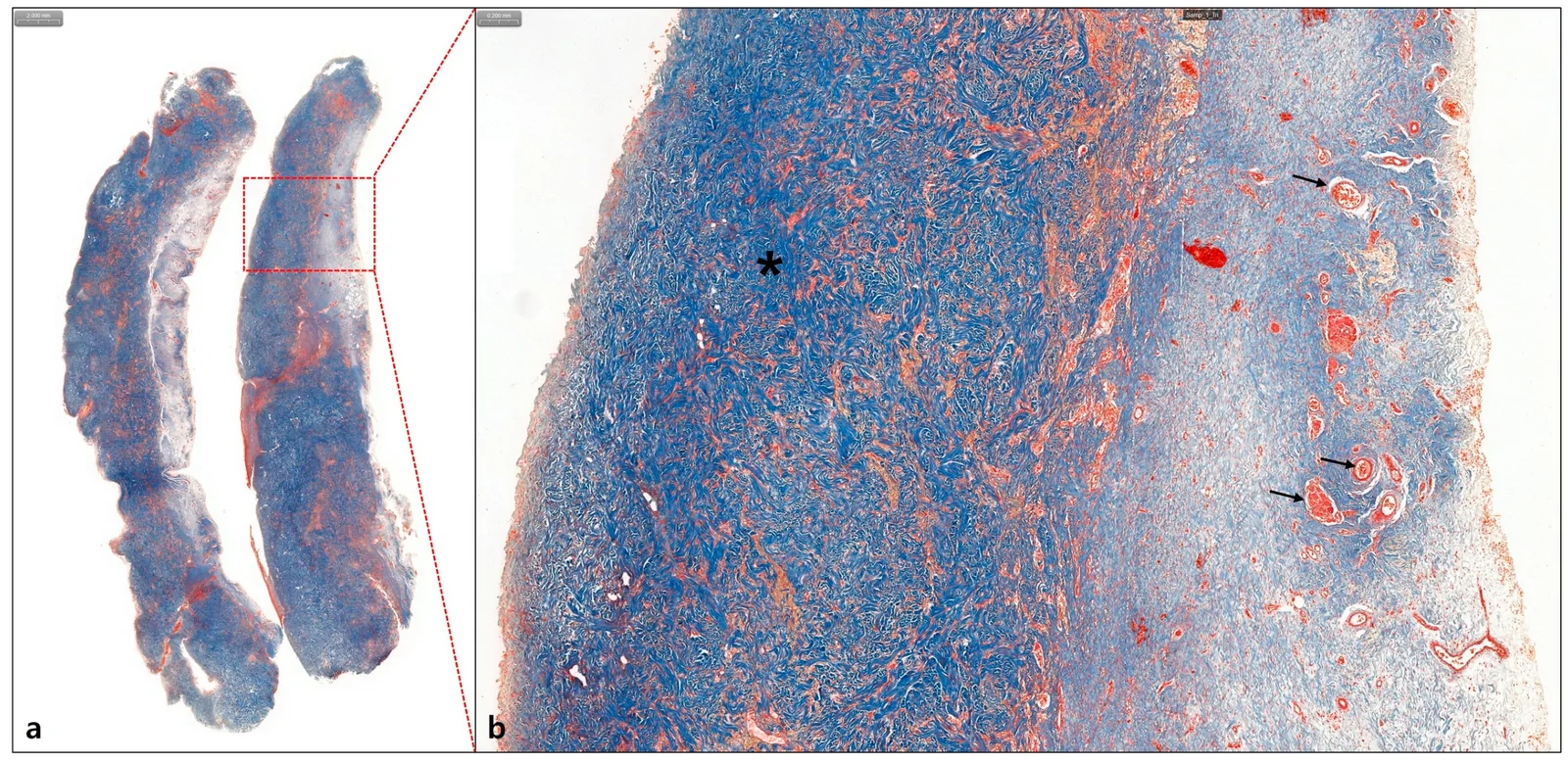
Masson trichrome histology of the excised envelope, sectioned in continuity from the skin surface to the periprosthetic capsule with the acellular dermal matrix in situ (collagen, blue; muscle and cytoplasm, red). (**a**) Whole-mount view of the full-thickness specimen showing diffuse replacement of the entire envelope by dense collagenous fibrosis; the boxed area is enlarged in (**b**). Scale bar, 2 mm. (**b**) Dense, hypocellular hyalinized collagen (asterisk) abutting a paler vascular zone containing congested, thick-walled vessels with mural hyalinization and concentric perivascular fibrosis (arrows), characteristic of chronic radiation vasculopathy. Scale bar, 0.2 mm. The findings illustrate the hypocellular, hypovascular substrate into which implant-based reconstruction was attempted.

Seven of the eight implants (87.5%) were removed, at 3.3, 7.6, 9.3, 10.0, 12.4, 12.5, and 51 months after reconstruction (Table 1), including one removal after a prior implant exchange. Failures occurred across the full range of radiotherapy-to-salvage intervals (1–24 years; Table 1): the earliest failure (3.3 months) followed an interval of 14 years, the latest (51 months) an interval of 11 years, and the patient irradiated 1 year before salvage surgery failed at 9.3 months; no relationship between the interval and the occurrence or timing of failure was apparent. All eight implant-based reconstructions were stand-alone prostheses placed directly beneath the irradiated mastectomy skin flaps, without vascularized flap coverage at the index operation. The eighth implant remains in place with a persistent 2-mm wound despite three corrective revisions to date and is followed as an at-risk retained implant; thus, no implant-based reconstruction in this series achieved complication-free success (0/8). Representative failure courses are shown in Figure 2, Figure 3, Figure 4 and Figure 5. Three failure patterns were observed. First, early envelope failure occurred in one patient who developed non-healing mastectomy skin-flap necrosis within 3 months and required salvage conversion to an LD flap (Figure 4), followed by two further implant exchanges and capsulotomy. Second, infection in the irradiated field occurred in one patient who developed periprosthetic infection with extensive destruction of the irradiated skin flap, and despite implant removal, performed without capsulectomy because the patient declined general anesthesia, required repeated wound revisions; at last follow-up, there was no seroma, and the residual defect was being managed with dressings while awaiting secondary healing. Third, late attritional failure occurred in one implant that survived for 51 months before persistent envelope inflammation (erythema, pain, and tightness without wound dehiscence, refractory to conservative management including allergy evaluation) necessitated explantation with capsulectomy after the patient declined further reconstruction (Figure 2 and Figure 3). In the patient converted to an LD flap, Masson trichrome histology of the full-thickness excised envelope demonstrated diffuse hypocellular fibrosis with mural hyalinization and concentric perivascular fibrosis of vessels in continuity from the dermis to the periprosthetic capsule, consistent with chronic radiation injury of the entire envelope (Figure 5).

### 3.4. Autologous Reconstruction

**Figure 6:**
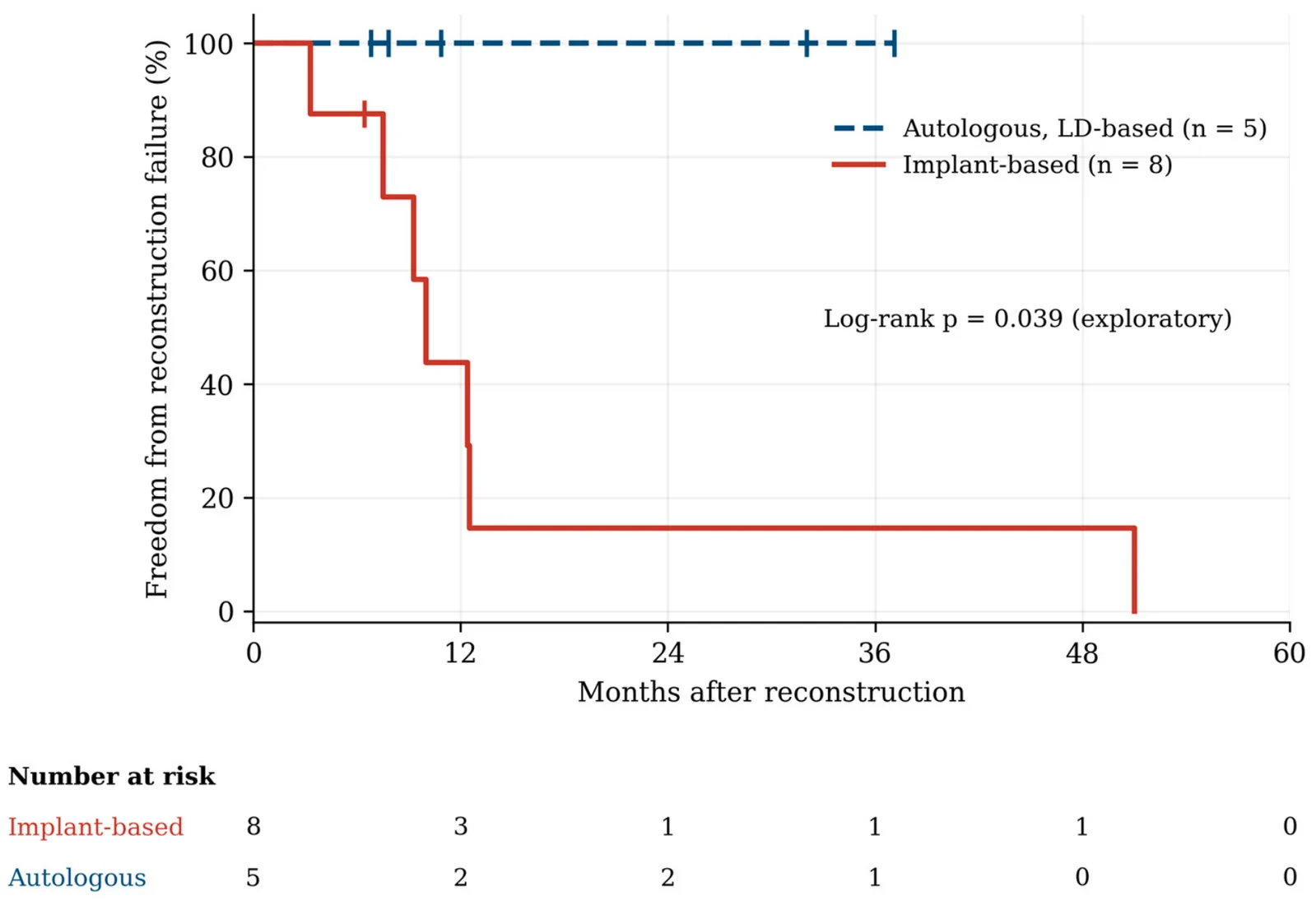
Kaplan–Meier estimates of freedom from reconstruction failure by reconstructive modality. Time at risk was measured from reconstruction to failure or, for reconstructions without failure, to last follow-up (data cutoff, 28 August 2026); tick marks indicate censored observations, and numbers at risk are shown below the plot. Log-rank *p* = 0.039 (exploratory). Because of the small number of patients, the curves are coarse, and only two autologous reconstructions were followed for more than 12 months.

All five autologous reconstructions healed completely. There were no reconstruction failures, no major wound complications, and no unplanned reoperations. Using the modality-neutral definition, reconstruction failure occurred in 7 of 8 implant-based and 0 of 5 autologous reconstructions (*p* = 0.005), and failure or major wound complication in 8 of 8 and 0 of 5, respectively (*p* = 0.0008) (Table 3, Figure 1); given the small sample, these *p* values are exploratory. The observed failure proportions were 87.5% (95% CI, 47.3–99.7%) for implant-based and 0% (95% CI, 0–52.2%) for autologous reconstruction, an absolute difference of 87.5 percentage points (95% CI, 32.0–97.8); for failure or major wound complication, the proportions were 100% (95% CI, 63.1–100%) and 0% (95% CI, 0–52.2%). These wide intervals indicate that, although the difference is large, its magnitude is imprecisely estimated. Kaplan–Meier estimates of freedom from reconstruction failure are shown in Figure 6. In the implant group, the estimated cumulative probability of failure was 56.3% (95% CI, 25.8–89.9%) at 12 months and 85.4% (95% CI, 52.9–99.3%) at 24 months, whereas no autologous reconstruction failed (log-rank *p* = 0.039). Time at risk was shorter in the autologous group, in which only two reconstructions had been followed for more than 12 months. In the sensitivity analysis restricted to the nine patients with at least 12 months of follow-up, failure occurred in 7 of 7 implant-based (100%; 95% CI, 59.0–100%) and 0 of 2 autologous reconstructions (0%; 95% CI, 0–84.2%; *p* = 0.028).

### 3.5. Descriptive Comparison with the Institutional Reference Cohort

Among the 689 implant-based reconstructions of breasts not previously irradiated at the time of reconstruction performed at our institution over the same period, 77 implants (11.2%) were removed: 20 of 144 (13.9%) in patients who received PMRT after reconstruction and 57 of 545 (10.5%) in patients who received no breast irradiation. The removal rate in the previously irradiated breast (7/8, 87.5%) was markedly higher than that in the whole reference cohort (11.2%; 95% CI, 8.9–13.8%; absolute difference, 76.3 percentage points; 95% CI, 41.6–86.8) and in the never-irradiated subgroup (10.5%; 95% CI, 8.0–13.3%) (both *p* < 0.001, unadjusted) (Table 4). Because the cohorts differ in indication, prior treatment, tissue characteristics, reconstructive technique, and follow-up, this difference is presented as descriptive context rather than as an adjusted or causal estimate of the effect of prior irradiation; because it rests on only eight study-cohort implants, it should be read as a point estimate with wide uncertainty rather than as a precisely determined effect.

## 4. Discussion

In this consecutive institutional series, implant-based total breast reconstruction of the previously irradiated breast after salvage mastectomy for IBTR showed a very high failure rate: 87.5% of implants were removed, the single retained implant harbors a persistent wound, and not one of eight implant reconstructions achieved complication-free success. In the same field, autologous reconstruction healed in every patient. Given a cohort of only thirteen, we regard this separation as a strong cautionary signal from a small consecutive institutional series rather than as definitive evidence; although the exploratory Fisher exact comparisons were significant, the estimates are imprecise (95% CI for the failure proportion, 47.3–99.7% with implant-based and 0–52.2% with autologous reconstruction) and may have been influenced by patient selection and surgical technique. The institutional reference cohort provides descriptive context: over the same period, implant reconstruction of breasts not previously irradiated at the time of reconstruction at our center carried an 11.2% removal rate, and 13.9% even with PMRT compared with 10.5% without breast irradiation; although these cohorts are not directly comparable and the comparison is unadjusted, the contrast with the salvage cohort is large. Even PMRT, the setting that dominates the literature on radiation and implant loss, was associated with only a modest absolute increase over the never-irradiated baseline, suggesting that reconstruction into an already-irradiated field may represent a distinct clinical problem.

The registry data also quantify how seldom reconstruction is undertaken in this setting: among at least 50 documented recurrences in previously irradiated, conserved breasts at our institution, fewer than one in five patients received immediate reconstruction at salvage mastectomy. This gap most plausibly reflects justified caution about operating within an irradiated field, and it is precisely the decision our algorithm is intended to inform, directing candidates toward autologous options rather than away from reconstruction altogether. This pattern is not specific to our center: in a recent Dutch multicenter cohort of 442 salvage mastectomies for IBTR, breast reconstruction was performed in only 19.7% [20], and in a recent Korean series, reconstruction after mastectomy for IBTR likewise remained the exception [21].

The mechanistic considerations that follow were not directly tested in this series; they are offered as a biologically plausible framework with which our clinical observations are consistent. Such a rationale can be drawn from three bodies of evidence: the effect of radiation on tissue, its effect on wound healing, and the behavior of an implant as a foreign body. Therapeutic irradiation injures normal tissue far beyond the acute phase. Endothelial damage progresses to obliterative endarteritis and capillary rarefaction; resident fibroblasts are depleted and driven into senescence; and sustained transforming growth factor-beta signaling establishes a self-perpetuating fibroatrophic process, so that the tissue becomes permanently hypovascular, hypocellular, and hypoxic (the classic three-H formulation) and continues to deteriorate rather than recover with time [9,10,11,12]. The primary lesion is vascular: capillaries are the most radiosensitive compartment, and endothelial injury proceeds through barrier disruption, oxidative stress, senescence, and a prothrombotic phenotype toward progressive microvascular rarefaction [22]. After whole-breast irradiation, this injury involves the entire skin envelope that must later cover any salvage reconstruction, in which a dose-dependent sequence of dermal fibrosis, appendage loss, and microvessel depletion is well characterized [23], and our observation of an implant failing after more than four years is consistent with a substrate whose reserve declines rather than plateaus [11]. Within this small dataset, however, failures occurred across the full range of radiotherapy-to-salvage intervals (1–24 years) without an apparent relationship to the interval (Section 3.3), so that a progressive, time-dependent effect, although plausible, could not be demonstrated here.

These changes would be expected to impair wound healing. Every phase of repair is compromised in irradiated tissue: the inflammatory response is dysregulated, fibroblast proliferation and collagen deposition are reduced, angiogenesis is blunted, and epithelialization is delayed, so that wounds that would close uneventfully in normal tissue instead dehisce, become chronic, or progress to necrosis [13,14,15]. A salvage mastectomy raises thin skin flaps entirely within such tissue and then asks them to heal over the additional tension of a reconstructed mound. Our first failure pattern, non-healing skin-flap necrosis and/or dehiscence within three months of implant placement, is consistent with this physiology, and the relentless revision course after infection in another patient is consistent with the limited capacity of irradiated tissue to re-establish healing once it has failed [14,15].

The implant itself compounds the problem as a foreign body. Classic experiments showed that the presence of foreign material lowers the inoculum of staphylococci required to establish infection by roughly four orders of magnitude [16], in part through a local defect of granulocyte function at the implant surface [17]. An avascular prosthetic surface additionally supports bacterial biofilm, which resists both host defenses and antibiotics [18] and subclinical biofilm colonization of breast-implant capsules is linked to capsular contracture [19]. In a normally vascularized pocket, these liabilities are usually tolerated; in an irradiated pocket that is hypoperfused, immunologically impaired, and mechanically fragile, the same foreign body is likely to be far less well tolerated. Our observation that the capsule perpetuated seroma and wound morbidity even after implant removal suggests that the entire foreign-body complex, not merely the prosthesis, may be the liability in irradiated tissue [19]. By contrast, an autologous flap imports non-irradiated, independently vascularized tissue that actively supports the compromised field, improves local perfusion, and adds no foreign surface; the uniform healing of our LD-based reconstructions, and the established reliability of flap reconstruction in irradiated fields are consistent with this [6,24].

Two secondary observations merit emphasis. First, the timing of failure was bimodal: six of seven failures occurred within 13 months, whereas one occurred beyond four years; early success therefore does not guarantee durability, and surveillance should not be abandoned. Second, the capsule itself appeared to perpetuate the foreign-body problem. In the two patients whose implants were removed without capsulectomy (in one to preserve the pocket for possible re-reconstruction, and in the other because the patient declined general anesthesia), the retained capsule in irradiated tissue was associated with recurrent seroma requiring outpatient aspiration in one and with a protracted wound-healing course in the other. When removal is undertaken in an irradiated field, we now favor completion capsulectomy whenever immediate autologous conversion is planned, and explicit counseling about retained-capsule morbidity whenever it is not. Multicenter data reinforce both points from the population side: salvage mastectomy is itself a morbid operation, with short-term complications in 45.2% of patients, and radiotherapy delivered after IBTR surgery independently doubles the odds of long-term complications (odds ratio, 2.14) [20], pertinent to the patient in our series in whom adjuvant irradiation followed the salvage reconstruction and chronic capsule-related seroma ensued.

The conduct of explantation itself deserves comment. In one recent failure (Figure 2 and Figure 3), although skin excision was kept to a minimum, closure after explantation and capsulectomy through the lateral inframammary access (the site of the original endoscopic mastectomy) proved unexpectedly difficult in the inelastic, irradiated envelope, and a local rotation flap with intensive wound care and intravenous alprostadil support was required to achieve stable coverage. When the implant is abandoned in an irradiated pocket, we therefore recommend performing explantation and capsulectomy through the incision alone, without excision of the skin flap, preserving every centimeter of the envelope for closure and for any future reconstruction.

These findings should be distinguished from the PMRT literature. In PMRT, radiation is delivered to a reconstructed breast. Meta-analyses and prospective cohorts consistently report increased complications and implant loss. Failure nevertheless remains far from universal, autologous reconstruction tolerates radiation better than implants, and sequencing strategies can partly mitigate risk [1,2,3,4,5,6]. The salvage-after-IBTR situation is different: the field is irradiated before any reconstruction exists, no sequencing strategy can protect the implant, and in our small series, the failure rate was very high. Reports specifically addressing implant reconstruction of the previously irradiated breast are sparse and heterogeneous: a highly selected two-stage series reported no failures [7], whereas a direct-to-implant series after salvage mastectomy reported wound dehiscence in 22% despite short follow-up [8]. Meta-analytic data confirm that prior irradiation roughly doubles to quadruples prosthetic failure relative to non-irradiated reconstruction [2,3]. We are aware of no prior series reporting so marked a separation between implant-based and autologous outcomes in this specific population; this observation, together with the proposed hypothesis-generating algorithm, constitutes the contribution of the present study.

The composition of the published IBTR-reconstruction series both supports this reading and explains why it has remained unrecognized. The large series reporting good outcomes after salvage mastectomy of the irradiated breast are overwhelmingly autologous or autologous-covered: in 111 consecutive immediate latissimus dorsi reconstructions for IBTR after breast-conserving therapy, postoperative complications occurred in 17.1% and implant loss in only 4.3%, although 82.9% of those implants lay beneath a freshly imported, non-irradiated LD flap [25]; in 60 skin-sparing mastectomies with immediate reconstruction for local recurrence, two-thirds were free abdominal flaps, no flap was lost, and only two of sixty reconstructions were implant-only [26]. An implant-dominant strategy shows the opposite signal: in a recent Korean series of 123 mastectomies for IBTR in which 73% of reconstructions were expander- or implant-based, reconstruction carried a significantly higher and more severe complication burden than mastectomy alone (18.8% vs. 4.0%; most complications Clavien–Dindo grade III) [21]. Read together with our data, these series suggest that what protects an implant in a previously irradiated field is coverage by imported, non-irradiated vascularized tissue; the stand-alone implant without flap coverage, the configuration of every failure in the present cohort, is precisely the configuration for which published data are scarcest and the risk appears greatest. This distinction refines the algorithm rather than contradicting it: an implant placed beneath an LD flap remains a legitimate autologous-based option in selected patients, whereas a stand-alone implant in an irradiated pocket should be understood as a different, far riskier operation.

Our results should also be weighed against series reporting more favorable outcomes with prosthetic reconstruction after salvage mastectomy. In a large single-surgeon experience, 121 carefully selected patients with prior breast-conserving therapy underwent immediate expander/implant reconstruction after salvage mastectomy and most completed reconstruction successfully, although mastectomy-flap necrosis was more than twice as frequent as in non-irradiated patients (18.0% vs. 7.7%) [27]; concurrent latissimus dorsi coverage has likewise been shown to facilitate implant-based reconstruction in this setting [28]. These reports indicate that implant-based reconstruction is not uniformly unsuccessful after salvage mastectomy and that patient selection and technique are critical. Several technical and selection factors in our series may have contributed to the high failure rate and cannot be separated from the effect of prior irradiation: seven of eight implant reconstructions followed nipple-sparing mastectomy, compared with one of five autologous reconstructions, and preoperative irradiation is an established predictor of complications after nipple-sparing mastectomy [29]; all implant reconstructions were stand-alone direct-to-implant reconstructions with an acellular dermal matrix, without vascularized flap coverage, so that the full prosthetic volume was placed beneath irradiated flaps at the index operation in seven of eight cases in the prepectoral plane without muscle coverage; the patient-level factors summarized in Table 2 and Supplementary Table S1 may have increased the risk in individual patients. Intraoperative mastectomy-flap perfusion was judged marginal in four implant patients who received alprostadil support; however, all four implant patients whose flaps appeared well perfused also experienced reconstruction failure, and only one patient was a smoker and one had diabetes, so neither marginal perfusion nor these comorbidities accounted for the overall failure pattern. Conversely, the autologous group had received, if anything, a higher median radiation dose, which argues against the possibility that flap patients were selected for less heavily irradiated tissue. Our data therefore do not establish that implant-based reconstruction is inappropriate for all previously irradiated patients; rather, they indicate that stand-alone prosthetic reconstruction beneath irradiated nipple-sparing or skin-sparing flaps carried a very high risk in our hands, and that the local condition of the irradiated tissue, which varies considerably between patients after breast-conserving therapy, should be central to the reconstructive decision, as reflected by the variation in outcomes across mastectomy and reconstruction types in other IBTR series [30]. Technical factors may well have contributed to some of these failures. Even so, the general selection criteria applied in this series (Section 2.3), which resemble those used by others [27], did not identify patients at acceptable risk, and the failure rate in this rare subgroup was markedly higher than that observed among all other implant-based reconstructions performed at our institution over the same period (Section 3.5). Although the small number of patients precludes robust statistical inference, these observations indicate that patient selection and the choice of reconstructive technique warrant considerably greater caution in women with a previously irradiated breast.

Treatment-selection and temporal biases also require consideration. Reconstructive modality was not randomized; it was chosen through surgeon–patient discussion (Section 2.3), and patients who accepted a donor-site scar may differ systematically from those who declined one. However, implant-based and autologous reconstructions were interspersed throughout 2022–2026 rather than separated into an early implant era and a later flap era: autologous reconstruction was already being used in 2023, and implant-based reconstruction was still performed in 2025 and 2026 (Table 1). The early failures therefore did not translate into an abrupt change in institutional practice within the study period, although our counseling has since changed: patients with a previously irradiated breast who seek total breast reconstruction after salvage mastectomy are now first offered, and encouraged to consider, autologous reconstruction, and implant-based reconstruction is considered, after thorough counseling, only for patients who are strongly apprehensive about or decline autologous surgery. In retrospect, under this approach, all eight implant patients would first have been offered autologous reconstruction; five of them (patients 6, 9, 10, 11, and 12) also had at least one additional factor that would now weigh further against a stand-alone implant (marginal intraoperative mastectomy-flap perfusion in four and, in one each, active smoking, diabetes, obesity [body mass index > 30 kg/m^2^], or radiotherapy after salvage surgery), whereas the remaining three (patients 3, 5, and 13) had no such factors apart from prior irradiation and nonetheless experienced reconstruction failure (Supplementary Table S1). A modest temporal effect on treatment selection nonetheless cannot be excluded.

It should also be emphasized that every autologous reconstruction in this series was latissimus dorsi-based (extended or boomerang LD flap) and that no abdominal free flap was performed. The inclusion of abdominal flaps in the proposed algorithm is therefore an extrapolation from external literature on autologous reconstruction after whole-breast irradiation and in irradiated fields [24,31,32], not an observation derived from this cohort. More generally, the clinically relevant contrast in our data may be less between “implant” and “autologous” reconstruction than between stand-alone prosthetic reconstruction and reconstruction that imports newly vascularized, non-irradiated tissue, a distinction supported by the low implant-loss rates reported when implants are placed beneath an LD flap [25,28].

**Figure 7:**
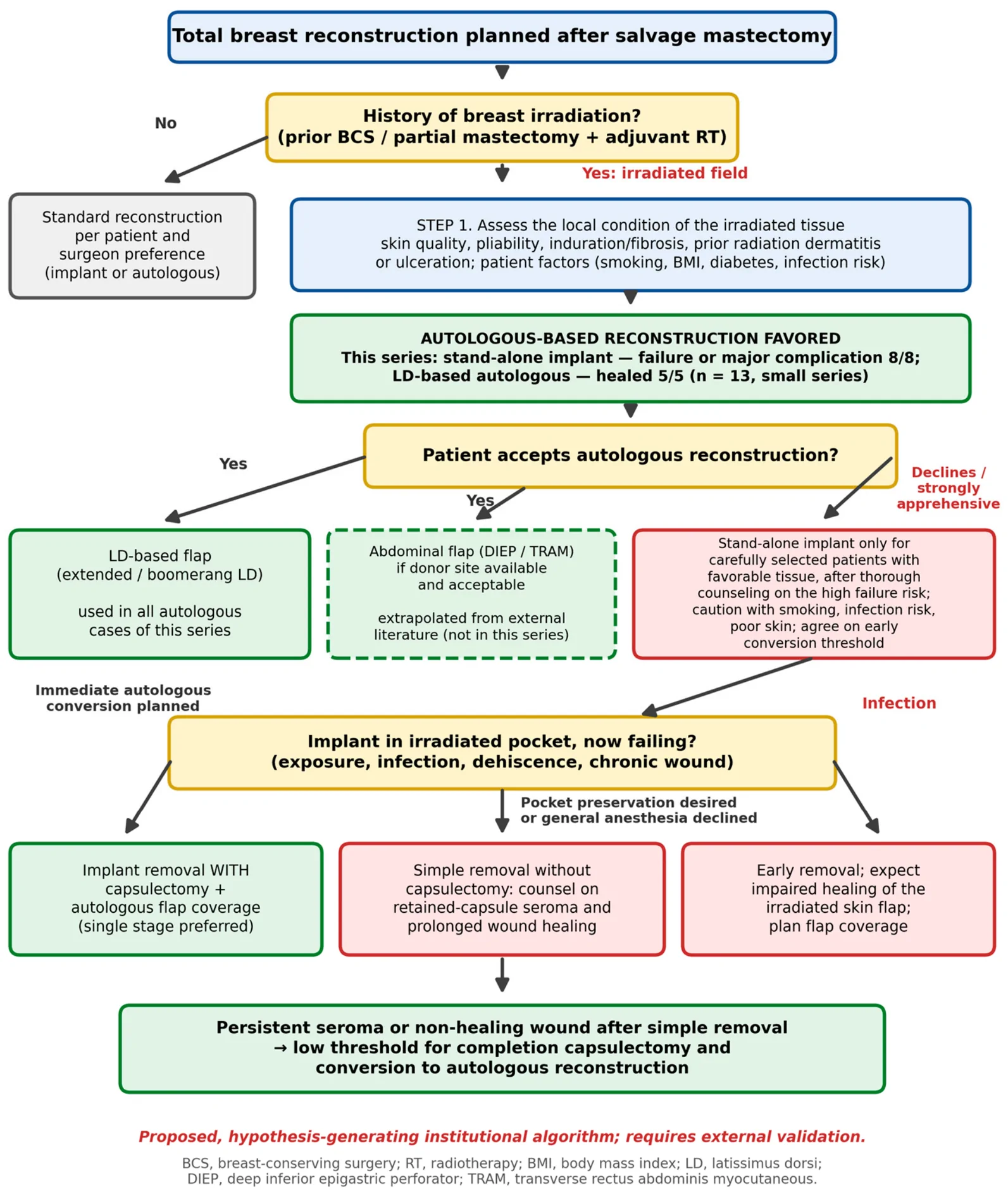
Proposed, hypothesis-generating autologous-first algorithm for total breast reconstruction after salvage mastectomy of the previously irradiated breast, including management of the failing implant and of the capsule. The abdominal flap option is extrapolated from external literature, as no abdominal flap was performed in this series; the algorithm requires external validation.

On the basis of these data, and with the caveats above, we propose the algorithm in Figure 7 as a hypothesis-generating institutional approach that requires external validation before wider adoption. After salvage mastectomy of a previously irradiated breast, assessment of the local condition of the irradiated tissue is the first step, and reconstruction incorporating well-vascularized autologous tissue is favored: an LD-based flap (extended or boomerang), as used in all autologous reconstructions in this series, or an abdominal flap when the donor site is available and acceptable, the latter option being extrapolated from external literature. Because every autologous reconstruction in this series was LD-based, this approach does not depend on microsurgical capability, an advantage due to the vascular biology itself, since pedicled transfer avoids microvascular anastomosis to irradiated recipient vessels, which carry increased thrombotic and anastomotic risk [22]. Stand-alone implant-based reconstruction may be considered for carefully selected patients with favorable tissue quality who decline autologous transfer after explicit disclosure of the high failure rate observed in this and other series, and should be approached with particular caution in the presence of additional risk factors such as infection risk, active smoking, or compromised skin quality. When an existing implant in an irradiated pocket is failing, the algorithm specifies management of the capsule according to whether immediate autologous conversion is planned, early aggressive removal in infection, and a low threshold for completion capsulectomy and autologous conversion when simple removal is followed by persistent seroma or a non-healing wound.

This study has limitations. It is retrospective and only included a single institution, and the cohort is small, reflecting the rarity of the clinical scenario; this rarity is itself a consequence of the efficacy of breast-conserving therapy, and no single institution should expect a large consecutive series. Although the observed differences were large, the estimates are imprecise, as reflected by the wide 95% CIs (for example, 0–52.2% for the autologous failure proportion), and the small sample precluded adjustment for smoking, body mass index, comorbidities, and other potential confounders. Group assignment was not randomized, selection factors cannot be excluded, and differences in mastectomy type (nipple-sparing mastectomy in seven of eight implant patients) and other technical factors may have contributed to the observed outcomes (Table 2). Radiotherapy parameters were verified against radiation oncology treatment archives by a radiation oncologist where retrievable, and were documented from outside-hospital records otherwise; all clinical dates and events were verified against source documents before the analyses reported here. Follow-up was shorter in the autologous group (median, 10.9 vs. 32.0 months), and two of the five autologous reconstructions had less than 8 months of follow-up; although most implant failures occurred within the first postoperative year and the Kaplan–Meier analysis accounts for the time at risk, later failures in the autologous group cannot be excluded, and follow-up of these patients and of the retained at-risk implant is ongoing. In addition, the institutional reference cohort was assembled by structured database query rather than chart-by-chart review; capture of all eight study-cohort implant patients by the query supports its sensitivity; denominators and PMRT status were subsequently verified by chart review; nevertheless, the reference cohort differs from the salvage cohort in several respects and is presented for descriptive context only. Registry variables describing primary treatment and prior irradiation derive from the keyword-based review of clinical notes and should be read as minimum estimates; recurrence status itself was physician-adjudicated, whereas radiotherapy documentation was incomplete, particularly for patients treated at outside hospitals. Patients undergoing risk-reducing mastectomy after previous breast-conserving therapy, such as BRCA1/2 carriers, share the problem of an irradiated envelope; they were nevertheless not included because the study was designed around oncologic salvage of IBTR (two patients in the present cohort, one BRCA1 and one BRCA2 carrier, were treated for IBTR, illustrating that the two populations overlap); whether our observations apply to that population warrants a separate study. Finally, our findings should be regarded as hypothesis-generating and require validation in larger, preferably multicenter, cohorts.

## 5. Conclusions

In the previously irradiated breast, after breast-conserving surgery, adjuvant radiotherapy, ipsilateral recurrence, and salvage total mastectomy, stand-alone implant-based reconstruction failed or caused major wound complications in every case in this small consecutive series, whereas reconstruction incorporating well-vascularized autologous tissue healed in every case. These findings represent a strong cautionary signal rather than definitive evidence. Our findings support an autologous-first approach in this setting, and the proposed algorithm should be regarded as a hypothesis-generating institutional approach that requires external validation.

## Figures and Tables

**Table 1:** Patient characteristics and outcomes (*n* = 13).

No.	Age (y)	Primary Treatment	Adjuvant RT (Dose/Fractions)	Salvage Mastectomy (Year, Type)	RT–Salvage Interval (y)	Reconstruction	Outcome	Course/Time to Failure	Follow-Up (mo)
1	43	BCS + SLNB + oncoplastic flap (2014)	50.4 Gy/28 fx + boost 10 Gy/5 fx	2026, simple	12	Extended LD flap	Healed	No failure	6.8
2	63	BCS + AD (2005)	50 Gy/25 fx + boost 10 Gy/5 fx (2006)	2025, SSM	19	Boomerang LD flap	Healed	No failure	10.9
3	49	BCS (2019, DCIS)	42.6 Gy/16 fx + boost 10 Gy/4 fx (2019)	2024, SSM	5	Implant	Removed	Removal at 12.5 mo	31.2
4	50	BCS + adj RT	50 Gy/25 fx + boost 16 Gy/8 fx (2019)	2023, SSM	4	Extended LD flap	Healed	No failure	32.0
5	51	BCS + adj RT	50.4 Gy/28 fx + boost 10 Gy/5 fx (2017)	2023, NSM	6	Implant	Removed	Removal + wound revision at 7.6 mo	32.8
6	67	BCS + SLNB (2018)	42.6 Gy/16 fx (IMRT) + boost 10 Gy/4 fx (2019)	2023, NSM	4	Implant	Removed	Exchange at 7.6 mo; removal at 10.0 mo	33.2
7	38	Excision ×2 (2017, DCIS); BRCA2 carrier	50 Gy/25 fx + boost 10 Gy/5 fx (outside institution, 2017)	2023, SSM	6	Extended LD flap	Healed	No failure	37.1
8	49	BCS + SLNB (2019)	42.56 Gy/16 fx (IMRT) + boost 15 Gy/6 fx	2026, NSM	7	Boomerang LD flap	Healed	No failure	7.8
9	55	BCS + adj RT (2011, outside)	50.4 Gy/28 fx, no boost (outside institution, 2011)	2022, NSM (endoscopic)	11	Implant	Removed	Removal at ~51 mo	52.1
10	47	PM + bilateral reduction (2022)	42.5 Gy/16 fx + boost 12 Gy/4 fx (54.5 Gy total, outside)	2026, NSM	4	Implant	Retained, at risk	Persistent 2-mm wound; three corrective revisions to date	6.4
11	53	BCS + adj RT (1998, outside); BRCA1 carrier	50 Gy/25 fx + boost 10 Gy/5 fx (outside institution, 1999)	2023, NSM	24	Implant	Removed	Simple removal without capsulectomy at 12.4 mo (pocket preserved for possible re-reconstruction); recurrent seroma managed by outpatient aspiration, ongoing	33.7
12	49	BCS + adj RT (2011)	50.4 Gy/28 fx + boost 10 Gy/5 fx (2011)	2025, NSM	14	Implant → LD salvage	Failed/converted	Skin-flap necrosis, non-healing → LD conversion at 3.3 mo; subsequent implant exchange ×2, capsulotomy	19.3
13	54	BCS + SLNB (2023)	40 Gy/15 fx + SIB boost 8 Gy (48 Gy total, 2024)	2025, NSM	1	Implant	Removed	Infection with extensive skin-flap damage; simple removal without capsulectomy at 9.3 mo (general anesthesia declined); repeated wound revisions; no seroma at last follow-up, residual defect managed with dressings	17.2

BCS, breast-conserving surgery; RT, radiotherapy; SLNB, sentinel lymph node biopsy; AD, axillary dissection; DCIS, ductal carcinoma in situ; PM, partial mastectomy; SSM, skin-sparing mastectomy; NSM, nipple-sparing mastectomy; LD, latissimus dorsi; IMRT, intensity-modulated radiotherapy; SIB, simultaneous integrated boost. Radiotherapy parameters were verified against radiation oncology treatment archives by a radiation oncologist or documented from outside-hospital records. RT–salvage interval, calendar years from the radiotherapy course preceding salvage surgery to salvage reconstruction. Follow-up, months from reconstruction to last follow-up (data cutoff, 28 August 2026); for failed reconstructions, the time to failure is given in the Course/Time to Failure column. BRCA1/2 status was not documented in the remaining patients.

**Table 2:** Baseline characteristics by reconstructive modality (descriptive comparison) ^1^.

Characteristic	Implant-Based (*n* = 8)	Autologous (*n* = 5)
Age at salvage reconstruction, years, median (range)	52 (47–67)	49 (38–63)
Primary ductal carcinoma in situ, *n*	1	1
Radiotherapy delivered at an outside institution, *n*	3	1
Hypofractionated whole-breast regimen, *n*	4	1
Tumor-bed boost delivered, *n*	7	5
Total radiotherapy dose, Gy, median (range)	53.6 (48.0–60.4)	60.0 (57.6–66.0)
Interval from radiotherapy to salvage reconstruction, years, median (range) ^2^	5.5 (1–24)	7 (4–19)
Mastectomy type, NSM/SSM/simple, *n*	7/1/0	1/3/1
Year of salvage reconstruction, range	2022–2026	2023–2026
Vascularized flap coverage at index operation, *n*	0	5
Documented BRCA1/2 pathogenic variant, *n*	1 (BRCA1)	1 (BRCA2)
Bilateral mastectomy at salvage surgery, *n*	2	0
Mastectomy specimen weight, g, median (range) ^3^	240 (111–673)	326 (68–463)
Direct-to-implant reconstruction, *n*	8	—
Implant volume, cc, median (range) ^3^	325 (130–600)	—
Acellular dermal matrix, *n*	8	1
Implant plane, prepectoral/subpectoral, *n*	7/1	—
Marginal mastectomy-flap perfusion with alprostadil support, *n* ^4^	4	1
Body mass index at salvage surgery, kg/m^2^, median (range)	22.0 (18.6–32.1)	24.7 (21.4–28.0)
Smoking, *n*	1	0
Diabetes mellitus, *n*	1	0
Chemotherapy before or after salvage surgery, *n*	4	3 (NR 2)
Radiotherapy after salvage surgery, *n*	1	1 (NR 2)
Follow-up after reconstruction, months, median (range) ^5^	32.0 (6.4–52.1)	10.9 (6.8–37.1)

NSM, nipple-sparing mastectomy; SSM, skin-sparing mastectomy. Values in the first nine rows are derived from Table 1. ^1^ Formal statistical testing was not performed given the sample size. ^2^ Calculated from the calendar year of the radiotherapy course preceding salvage surgery to the year of salvage reconstruction. ^3^ For bilateral salvage mastectomy, the value for the previously irradiated side is shown. ^4^ Mastectomy-flap perfusion was assessed clinically in all patients. ^5^ To 28 August 2026. NR, not recorded. BRCA1/2 status was not documented in the remaining patients. Reconstructive details for each patient are provided in Supplementary Table S1.

**Table 3:** Reconstruction outcomes by modality.

Outcome	Implant-Based (*n* = 8)	Autologous (*n* = 5)	*p* Value ^1^
Reconstruction failure (modality-neutral) ^2^	7 (87.5%)	0	0.005
95% CI, %	47.3–99.7	0–52.2	
Reconstruction failure or major wound complication	8 (100%)	0	0.0008
95% CI, %	63.1–100	0–52.2	
Complication-free success	0	5 (100%)	N/A
Unplanned reoperation	8 (100%)	0	0.0008
Cumulative failure at 12/24 months (Kaplan–Meier), %	56.3/85.4	0/0	0.039 ^3^
Reconstruction failure, patients with ≥12 months of follow-up	7/7 (100%)	0/2	0.028
Follow-up after reconstruction, months, median (range) ^4^	32.0 (6.4–52.1)	10.9 (6.8–37.1)	N/A
Time to removal or conversion, months	3–51 (6 of 7 within 13 mo)	N/A	N/A

^1^ Fisher exact test, two-sided. Major wound complication: persistent wound, skin-flap necrosis, periprosthetic infection, or chronic seroma. ^2^ Prosthetic reconstruction: definitive implant removal or unplanned conversion to flap reconstruction; autologous reconstruction: total flap loss, flap removal, or unplanned conversion to another reconstructive method. ^3^ Log-rank test. ^4^ To last follow-up (data cutoff, 28 August 2026). 95% CIs for proportions are exact (Clopper–Pearson).

**Table 4:** Implant removal by irradiation setting (descriptive comparison with the institutional reference cohort).

Setting of Implant-Based Reconstruction	*n*	Implant Removal, *n* (%) [95% CI]	*p* vs. Previously Irradiated ^1^
Previously irradiated breast (salvage after BCS/PM + RT, study cohort)	8	7 (87.5) [47.3–99.7]	N/A
Not previously irradiated at reconstruction, all (institutional reference cohort)	689	77 (11.2) [8.9–13.8]	<0.001
PMRT after reconstruction	144	20 (13.9) [8.7–20.6]	<0.001
No breast irradiation	545	57 (10.5) [8.0–13.3]	<0.001

^1^ Fisher exact test, two-sided, versus the previously irradiated study cohort. BCS, breast-conserving surgery; PM, partial mastectomy; RT, radiotherapy; PMRT, postmastectomy radiotherapy. The reference cohort was identified by structured database query (validated against all eight study-cohort implant patients) with removal ascertained from the institutional removal registry and verified by chart review. Comparisons are unadjusted and descriptive. 95% CIs are exact (Clopper–Pearson).

## Data Availability

The data presented in this study are available on request from the corresponding author owing to patient privacy and institutional restrictions.

## Supplementary Materials

The following supporting information can be downloaded at: https://www.mdpi.com/article/10.3390/cancers18203409/s1, Table S1: Reconstructive technique and patient-level risk factors for each patient.
