# Supplementary material for: Implant-Based Versus Autologous Reconstruction After Salvage Mastectomy for Ipsilateral Breast Tumor Recurrence in the Previously Irradiated Breast: A Consecutive Institutional Series and a Proposed Autologous-First Algorithm
Source: Cancers (Basel). 2026 Oct 21;18(20):3409. doi: 10.3390/cancers18203409 (PMC13702566; doi:10.3390/cancers18203409)
Supplement: Supplementary file 1 [file cancers-18-03409-s001.zip › cancers-4605628-supplementary.pdf]

Table S1. Per-patient reconstructive technique and patient-level risk factors (n = 13).

Pt	Recon.	Side	Mastectomy (date)	Incision	DTI / TE	Implant plane	ADM	Implant / flap	Specimen (g)	Mastectomy-flap perfusion assessment	Intraoperative mastectomy-flap perfusion	BMI	Smoking	DM	Chemo	RT after salvage	BRCA1/2	Outcome
1	Ext. LD	L	Simple (2026-02-02)	Previous rotation-flap scar	—	—	No	Flap 5×15 cm	68	Clinical	Good	22.2	No	No	NR	NR	ND	Healed
2	Boom. LD	L	SSM (2025-10-01)	Circumareolar (NAC excision)	—	—	No	Flap 380 g	326	Clinical	Good	24.7	No	No	Post	Yes	ND	Healed
3	Implant	R	SSM (2024-01-22)	Nipple-to-axilla oblique (NAC excision)	DTI	Prepectoral	Myderm 16×16	Mentor SRM 130 cc	151	Clinical	Good	20.9	No	No	No	No	ND	Removed 12.5 mo
4	Ext. LD	R	SSM (2023-12-27)	Nipple-to-axilla oblique (NAC excision)	—	—	No	Flap 360 g	463	Clinical	Good	28.0	No	No	Pre	No	ND	Healed
5	Implant	R	NSM (2023-12-04)	Nipple-to-axilla oblique	DTI	Prepectoral	Myderm 18×18	Mentor MC 215 cc	294	Clinical	Good	21.9	No	No	Pre	No	ND	Removed + revision 7.6 mo
6	Implant	L	NSM (2023-11-22)	Nipple-to-axilla oblique	DTI	Prepectoral	Myderm 20×20	Mentor SRM 300 cc	340	Clinical; alprostadil	Marginal	23.8	No	Yes	Pre	No	ND	Exchange 7.6 mo; removed 10.0 mo
7	Ext. LD	L	SSM (2023-07-26)	Oblique with partial skin excision	—	—	Megaderm 6×12	Flap 445 g	416	Clinical	Good	25.3	No	No	Pre + post	No	BRCA2	Healed
8	Boom. LD	L	NSM (2026-01-02)	Nipple-to-axilla oblique	—	—	No	Flap 4×16, 6×18 cm	233	Clinical; alprostadil	Marginal	21.4	No	No	NR	NR	ND	Healed
9	Implant	R*	Bilateral endoscopic NSM (2022-04-26)	Lateral inframammary (endoscopic)	DTI	Prepectoral	Megaderm 20×20	Eurosilicone 350 cc	260*	Clinical; alprostadil	Marginal	22.1	No	No	Post	No	ND	Removed ~51 mo
10	Implant	R	NSM (2026-02-13)	IMF (previous reduction scar)	DTI	Subpectoral	SCDerm 20×20	Mentor MP 600 cc	673	Clinical; alprostadil	Marginal	32.1	No	No	No	No	ND	Retained; persistent 2-mm wound
11	Implant	L*	Bilateral NSM + Rt ALND (2023-11-07)	Nipple-to-axilla oblique	DTI	Prepectoral	Megaderm 20×20	Mentor MP 450 cc*	220*	Clinical (blanching)	Good	23.3	No	No	Post	Yes	BRCA1	Removed 12.4 mo (no capsulectomy); recurrent seroma
12	Implant	R	NSM (2025-01-17)	Nipple-to-axilla oblique	DTI	Prepectoral	SCDerm 20×20	Mentor HP 550 cc	120	Clinical; alprostadil	Marginal	19.7	Yes	No	No	No	ND	Necrosis; LD conversion 3.3 mo
13	Implant	R	NSM (2025-03-21)	Nipple-to-axilla oblique	DTI	Prepectoral	SCDerm 20×20	Mentor MP 225 cc	111	Clinical (blanching)	Good	18.6	No	No	No	No	ND	Infection; removed 9.3 mo (no capsulectomy)

Pt, patient (numbered as in Table 1); Recon., reconstruction; Ext. LD, extended latissimus dorsi flap; Boom. LD, boomerang latissimus dorsi flap; SSM, skin-sparing mastectomy; NSM, nipple-sparing mastectomy; ALND, axillary lymph node dissection; NAC, nipple–areola complex; IMF, inframammary fold; DTI, direct-to-implant; TE, tissue expander; ADM, acellular dermal matrix; SRM, smooth round moderate; MC, moderate classic; MP, moderate plus; HP, high profile; BMI, body mass index (kg/m²) at salvage surgery; DM, diabetes mellitus; Chemo, chemotherapy before (Pre) and/or after (Post) salvage surgery; RT, radiotherapy; ND, not documented; NR, not recorded; —, not applicable. Specimen, mastectomy specimen weight. Mastectomy-flap perfusion was assessed clinically (skin color and capillary refill on blanching); intravenous alprostadil was given when intraoperative perfusion appeared marginal. Times are from reconstruction to removal or conversion. All implant-based reconstructions were stand-alone prostheses without vascularized flap coverage at the index operation. * Bilateral salvage mastectomy; values for the previously irradiated side are shown.
